# *In Silico* Design for Adenosine Monophosphate-Activated Protein Kinase Agonist from Traditional Chinese Medicine for Treatment of Metabolic Syndromes

**DOI:** 10.1155/2014/928589

**Published:** 2014-05-11

**Authors:** Hsin-Chieh Tang, Calvin Yu-Chian Chen

**Affiliations:** ^1^Department of Biomedical Informatics, Asia University, Taichung 41354, Taiwan; ^2^Department of Medicine, China Medical University, Taichung 40402, Taiwan

## Abstract

Adenosine monophosphate-activated protein kinase (AMPK) acts as a master mediator of metabolic homeostasis. It is considered as a significant millstone to treat metabolic syndromes including obesity, diabetes, and fatty liver. It can sense cellular energy or nutrient status by switching on the catabolic pathways. Investigation of AMPK has new findings recently. AMPK can inhibit cell growth by the way of autophagy. Thus AMPK has become a hot target for small molecular drug design of tumor inhibition. Activation of AMPK must undergo certain extent change of the structure. Through the methods of structure-based virtual screening and molecular dynamics simulation, we attempted to find out appropriate small compounds from the world's largest TCM Database@Taiwan that had the ability to activate the function of AMPK. Finally, we found that two TCM compounds, eugenyl_beta-D-glucopyranoside and 6-O-cinnamoyl-D-glucopyranose, had the qualification to be AMPK agonist.

## 1. Introduction

One study has found that the cell “starvation” signal transduction pathway reveals the process of cell “starvation” signal transduction mechanisms. This finding is considered as a significant millstone to treat metabolic syndromes including obesity, diabetes, and fatty liver [1, 2]. Adenosine monophosphate-activated protein kinase (AMPK) was always a hot issue in the recent 10 years because it plays an essential role in sensing cellular energy or nutrient status [3, 4]. AMPK exists in several organs including skeletal muscles, brain, and liver [5]. For example, when skeletal muscles have exercised for a period of time, activated AMPK helps the cells adapt to energy challenge by increasing glucose uptake [6]. AMPK acts as a master mediator of metabolic homeostasis [7]. The effect of AMPK activation is switching on the catabolic pathways consisting of cellular glucose uptake, glycolysis, fatty acid oxidation, inhibition of triglyceride, cholesterol, and protein synthesis [8, 9]. The upstream signal molecule, such as adiponectin, stimulates AMPK to utilize glucose and fatty acid [10]. The activated AMPK in the hypothalamus increases the appetite or desire for food intake [11, 12]. Adiponectin stimulates AMPK to protect the heart against myocardial ischemia [13, 14]. Another upstream signal molecule, leptin, activates AMPK to stimulate fatty acid oxidation [15]. AMPK in the hypothalamus mediates thyroid hormone to regulate energy balance, too [16].

AMPK consists of three subunits, *α*, *β*, and *γ* [17]. Each of the subunits has a specific structure and function [18, 19]. The following mechanism explains how the three subunits work. When the cells need energy, adenosine triphosphate (ATP) converts to adenosine diphosphate (ADP) and AMP by releasing energy simultaneously. At the normal condition, excess AMP binds to the *γ* subunit of AMPK [20]. After binding of *γ* subunit exposes the active site, Thr172, on the catalytic *α* subunit of AMPK [21]. For all of the three subunits, *α* subunit is the most important. Activation of AMPK follows conformational change of *α* subunit and phosphorylation at Thr172 [22]. The phosphorylation is induced by the upstream signal molecule such as liver kinase B1 (LKB1) [23]. The *β* subunit is located between *α* and *γ* subunits and is associated with the function of glycogen sensor [24]. ADP can also bind to *γ* subunit and protect AMPK from dephosphorylation but cannot cause conformational change [25].

Physiological study has approved that blood glucose is partly regulated by AMPK [26]. Activating AMPK by its agonist decreases insulin resistance induced by obesity [27]. Drug design for AMPK activator provides the hope for treating type 2 diabetes mellitus [28]. AMPK has become the drug target for managing diabetes and metabolic syndrome [29]. Metformin is the classic antidiabetes medicine involved in activation of AMPK [30, 31]. Investigation of AMPK has new findings recently. Activated AMPK has the function of anti-inflammation by a mimic state of pseudostarvation [32]. AMPK can regulate cell growth via signal integration [33]. The LKB1-AMPK pathway can mediate cellular autophagy or apoptosis by phosphorylating p27kip1 [34]. Thus the LKB1-AMPK pathway has been approved in the field of tumor suppression [35]. AMPK inducing phosphorylation of Unc-51-like kinase 1 (ULK1) can cause autophagy in an energetic exhaustion status. ULK1 is a serine/threonine-protein kinase [36, 37].

Due to progress of modern technology, the binding phenomena of protein dynamics motion and structure changing can be analyzed by computational simulation [38, 39].* In silico* investigation of biology or computational systems biology helps us to explore the protein-protein or protein-molecule interaction [40, 41]. These methods make it possible to participate in computer-aided drug design (CADD) [42, 43]. CADD techniques save our time to select appropriate drug compound rapidly compared with traditional one-by-one biochemistry [44, 45]. CADD procedures consist of virtual screening, validations, and analysis [46, 47]. Virtual screening and analysis employ docking and molecular dynamics (MD) simulation [48–50]. MD can predict how long the compound needs to form stable complex structure with target protein [51]. A series of statistics or score systems facilitate docking and MD accuracy [52]. Best candidates from virtual screening and MD can be selected as potential therapeutic drugs [53].

With progress in medical technology, many diseases can be resolved nowadays [54–56]. When we know how the diseases happen, we can handle them in smart ways [57–59]. Because of coordinating cell growth and autophagy, AMPK has become an emerging target for drug design of tumor inhibition [60]. Design of small molecular AMPK agonist is possible [61]. Small molecular drug design by CADD has been applied in systems biology extensively [62–64]. Traditional Chinese medicine (TCM) has therapeutic effect on many diseases [65–68]. In this study, we attempted to find out appropriate small compounds from the world's largest TCM Database@Taiwan that had the ability to activate the function of AMPK [69].

## 2. Materials and Methods

### 2.1. Compound Database

To identify potential AMPK activators from TCM, we downloaded all small molecules from TCM Database@Taiwan (http://tcm.cmu.edu.tw/) to carry on AMPK agonist screening [69].

### 2.2. Homology Modeling

The AMPK protein sequence was obtained from the Uniprot Knowledgebase (Q13131, human). The 3D structure of rat AMPK was obtained from Protein Data Bank (PDB ID: 2Y94). The sequence of human AMPK (Q13131) and homologous crystal structure of rat AMPK (2Y94) were aligned by the modeler mode in Accelrys Discovery Studio (DS) 2.5. The identity and similarity can be calculated according to the result of sequence alignment. Homology modeling of AMPK was established by the Build Homology Models protocol in DS 2.5. The reasonable AMPK model was further validated by Ramachandran plot with Rampage protocol and Verify score with Profiles-3D protocol in DS 2.5.

### 2.3. Structure-Based Virtual Screening

The ligands from TCM Database@Taiwan and the control ligand, adenosine monophosphate (AMP), were prepared for specified techniques. Ligand docking was performed by the LigandFit module in DS 2.5. The force field of Chemistry at HARvard Molecular Mechanics (CHARMm) was utilized to minimize all docking poses [70]. Absorption, distribution, metabolism, excretion, and toxicity (ADMET) were applied for all TCM compounds [71]. The scoring systems such as Dock score, piecewise linear potentials (PLP), and potential of mean force (PMF) were calculated by the LigandFit module in DS 2.5 [72, 73].

### 2.4. Disorder Prediction

We drew disorder disposition to exclude disordered residues by the program of PONDR-FIT in the DisProt website [74, 75].

### 2.5. Molecular Dynamics (MD) Simulation

The package of GROMACS (GROningen MAchine for Chemical Simulations) was used for MD simulation. We employed SwissParam to determine topology and parameters of small compounds for GROMACS simulation. The cytoplasmic condition was set with transferable intermolecular potential 3P (TIP3P) water at 0.9% sodium chloride concentration. After docking, selected protein-ligand complexes were conducted under the following phases: minimization, heating, equilibration, and production. The minimization protocol included 500 steps of steepest descent and 500 steps of conjugated gradient. The heating time was 50 ps from 50 K to 310 K. The equilibration time was 150 ps at 310 K. The production time was 5000 ps with constant temperature dynamics method. The time for temperature decay was 0.4 ps. We utilized the trajectory analysis to illustrate root mean square deviation (RMSD), Gyrate, solvent accessible surface (SAS), root mean square fluctuation (RMSF), total energy, database of secondary structure assignment (DSSP), matrices of smallest distance of residues for individual ligands, and protein during MD. We calculated the formation and distance of hydrogen bond (H-bond), too. Best distance of H-bond was set at 0.3 nm.

### 2.6. Ligand Pathway

To analyze the ligand pathway, we employed the software of LigandPath module to illustrate the possible pathway of each ligand. A surface probe and minimum clearance were set at 6 Å and 3 Å, respectively.

## 3. Results

### 3.1. Homology Modeling

**Figure 1:**
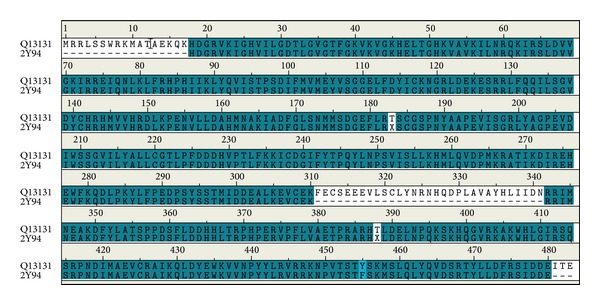
Sequence alignment between Q13131 human and template (2Y94). The identity is 89.4% and the similarity is 89.6%.

**Figure 2:**
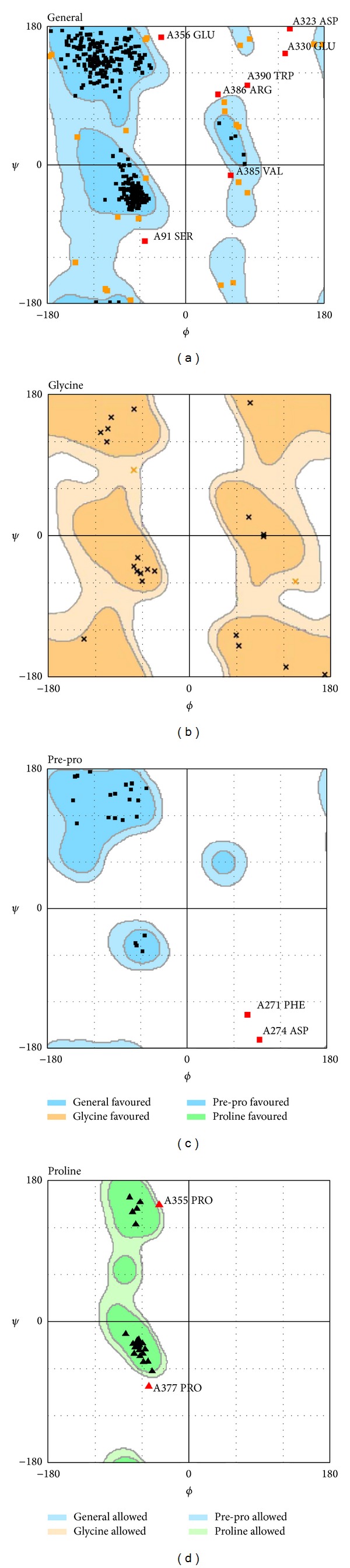
Ramachandran plot of AMPK-modeled structure. Number of residues in favored region (~98.0% expected) is 422 (91.7%). Number of residues in allowed region (~2.0% expected) is 27 (5.9%). Number of residues in disallowed region is 11 (2.4%).

**Figure 3:**
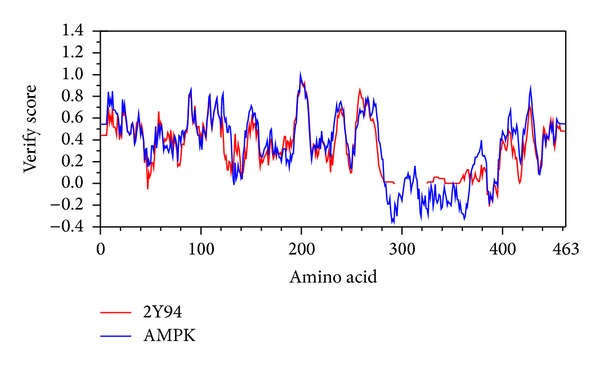
Verify score of AMPK-modeled structure. Most residues are positive values except residues from 290 to 360.

According to sequence alignment between Q13131_human and template (2Y94), the overall identity was 89.4% and similarity was 89.6% (Figure 1). Ramachandran plot of AMPK-modeled structure showed that 91.7% of residues were in the favored area, 5.9% were in the allowed area, and only 2.4% were in the disallowed area (Figure 2). The Verify score showed that most residues were positive values except residues from 290 to 360 (Figure 3).

### 3.2. Structure-Based Virtual Screening

**Figure 4:**
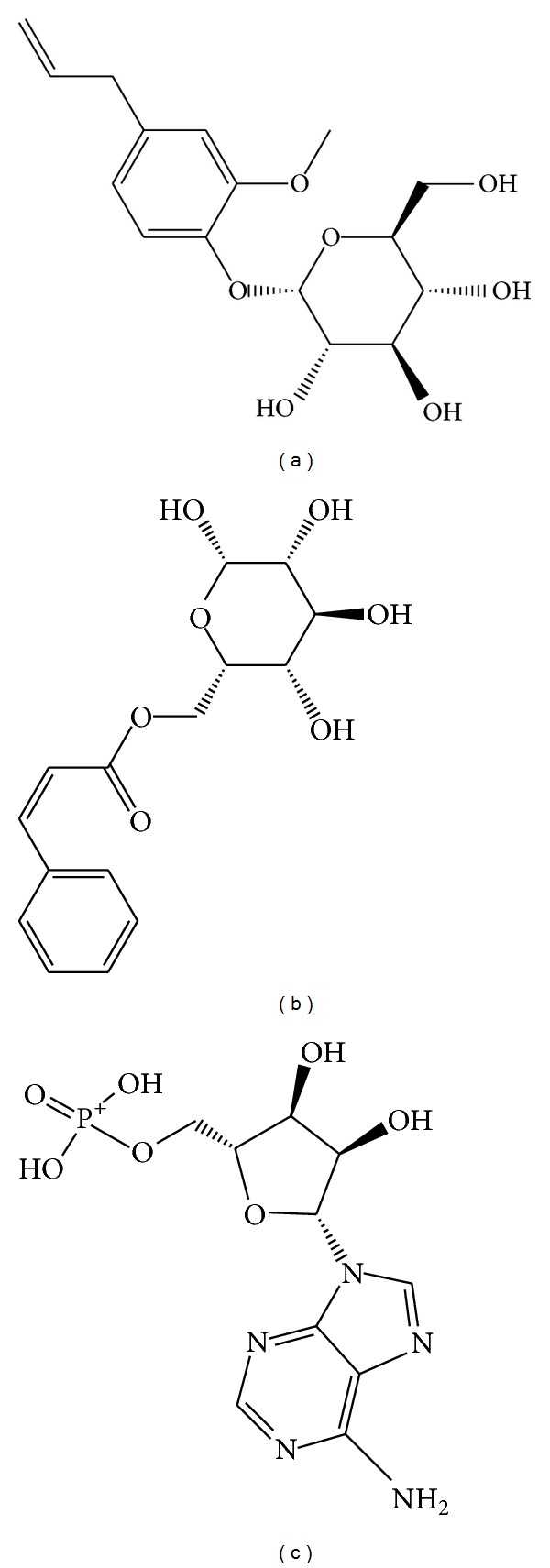
Scaffold of top 2 candidate compounds: (a) eugenyl_beta-D-glucopyranoside, (b) 6-O-cinnamoyl-D-glucopyranose, and the control (c) AMP.

**Figure 5:**
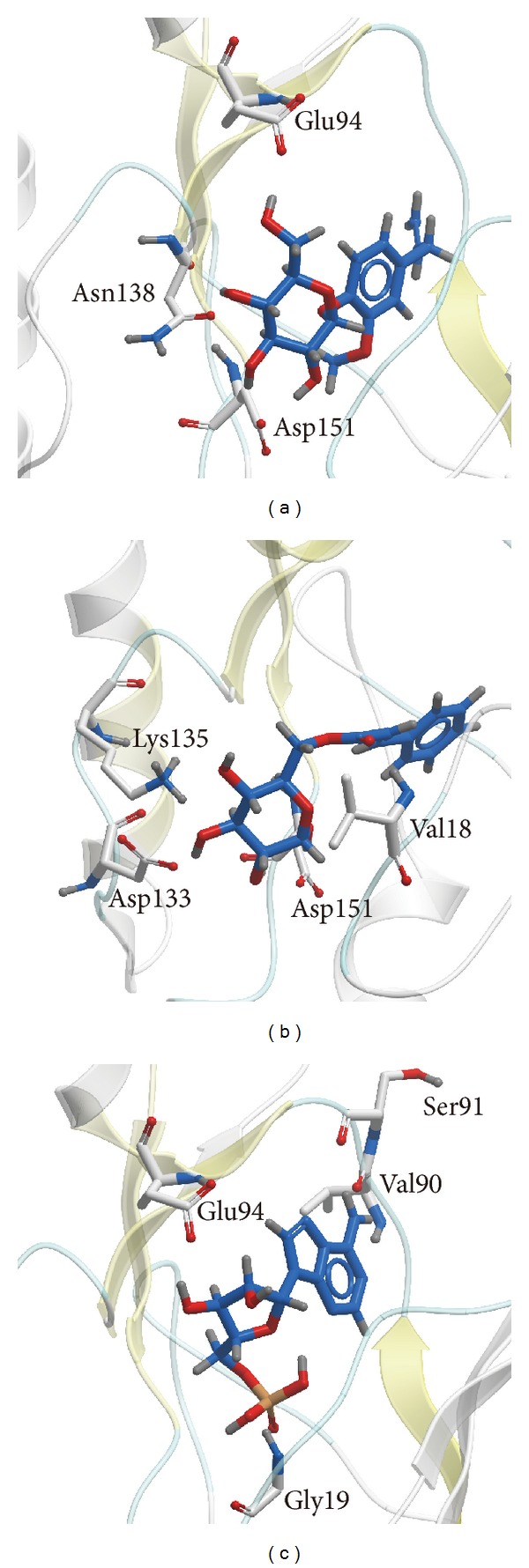
Docking poses of (a) eugenyl_beta-D-glucopyranoside, (b) 6-O-cinnamoyl-D-glucopyranose, and (c) AMP. The common residue is ASP151.

Table 1 showed absorption level of ADMET, Dock score, PLP1, PLP2, and PMF of the top 7 TCM compounds ranked by absorption level of ADMET. By the integration of these data, we selected the first 2 compounds, eugenyl_beta-D-glucopyranoside and 6-O-cinnamoyl-D-glucopyranose, as candidates for further survey (Figure 4). Docking poses of eugenyl_beta-D-glucopyranoside, 6-O-cinnamoyl-D-glucopyranose, and the control (AMP) with AMPK were illustrated in Figure 5. Eugenyl_beta-D-glucopyranoside formed H-bond with Glu94, Asn138, and Asp151 (Figure 5(a)). 6-O-Cinnamoyl-D-glucopyranose formed H-bond with Val18, Asp133, Lys135, and Asp151 (Figure 5(b)). AMP formed H-bond with Gly19, Val90, Ser91, and Glu94 (Figure 5(c)).

### 3.3. Disorder Prediction

**Figure 6:**
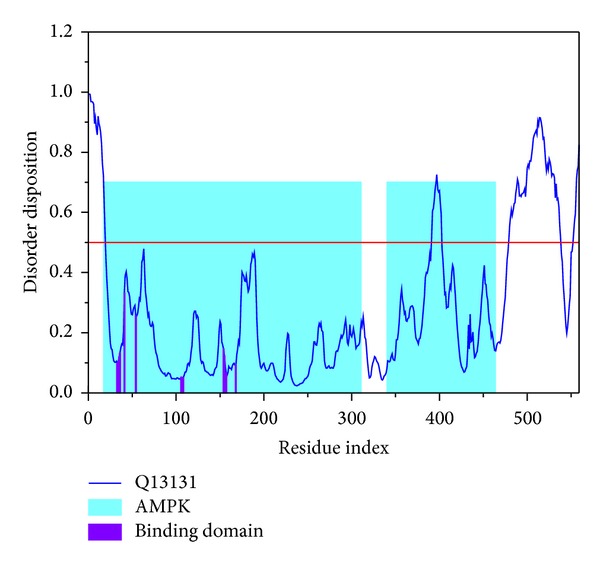
Disorder disposition of AMPK-modeled structure. Main residues (Val18 to ASP151) are in the nondisordered region (below the red line).

Main residues (Val18 to Asp151) of AMPK-modeled structure for the 2 candidates and the control were not in the disordered area, so there was no influence to the shape of the binding site (Figure 6).

### 3.4. Molecular Dynamics (MD) Simulation

**Figure 7:**
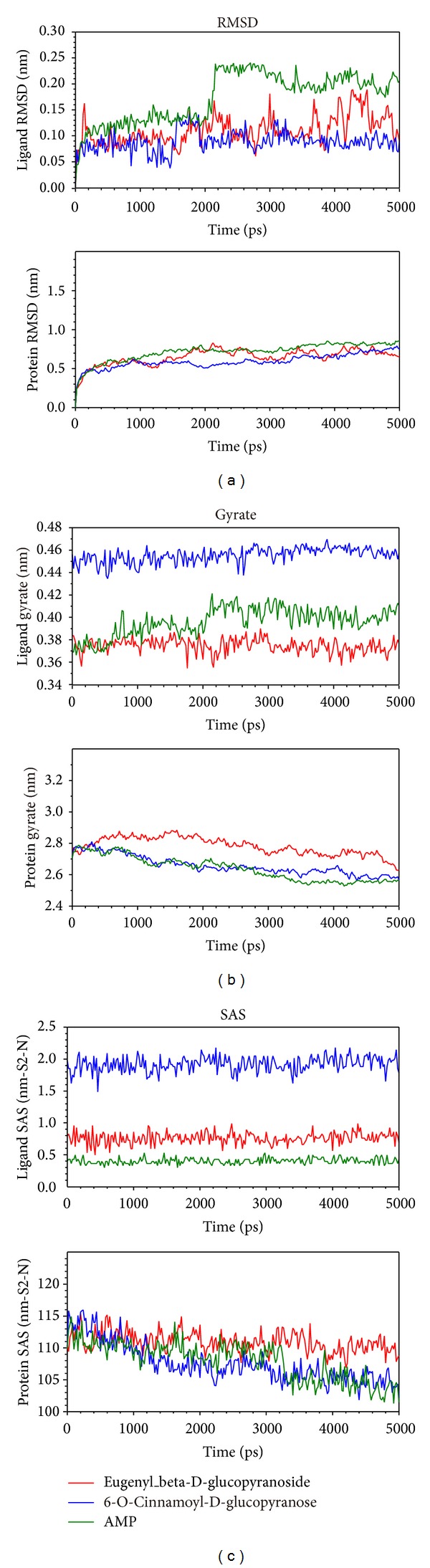
Ligand and protein (a) root mean square deviation (RMSD), (b) Gyrate, and (c) solvent accessible surface (SAS).

MD trajectories generated by GROMACS were illustrated. We utilized root mean square deviation (RMSD) to show the deviation degree of each ligand or protein from the beginning to the end of MD. When the ligand formed a complex with the protein (AMPK), eugenyl_beta-D-glucopyranoside, 6-O-cinnamoyl-D-glucopyranose, and the control (AMP) had first larger deviations at 1600 ps, 1450 ps, and 2000 ps, respectively. AMP had a larger average deviation than the candidates (Figure 7(a)). We utilized Gyrate to measure the average distance of the atoms to the center of each mass. In other words, Gyrate showed the compact degree of each ligand or protein. 6-O-Cinnamoyl-D-glucopyranose had the largest ligand Gyrate values, but eugenyl_beta-D-glucopyranoside had the largest protein Gyrate values. All the proteins had a trend to compact status at the end of MD (Figure 7(b)). We utilized solvent accessible surface (SAS) to measure the surface area of each ligand or protein in contact with the water. 6-O-Cinnamoyl-D-glucopyranose had the largest ligand SAS values, but eugenyl_beta-D-glucopyranoside had the largest protein SAS values. All the proteins had a trend to decreased SAS value at the end of MD (Figure 7(c)).

**Figure 8:**
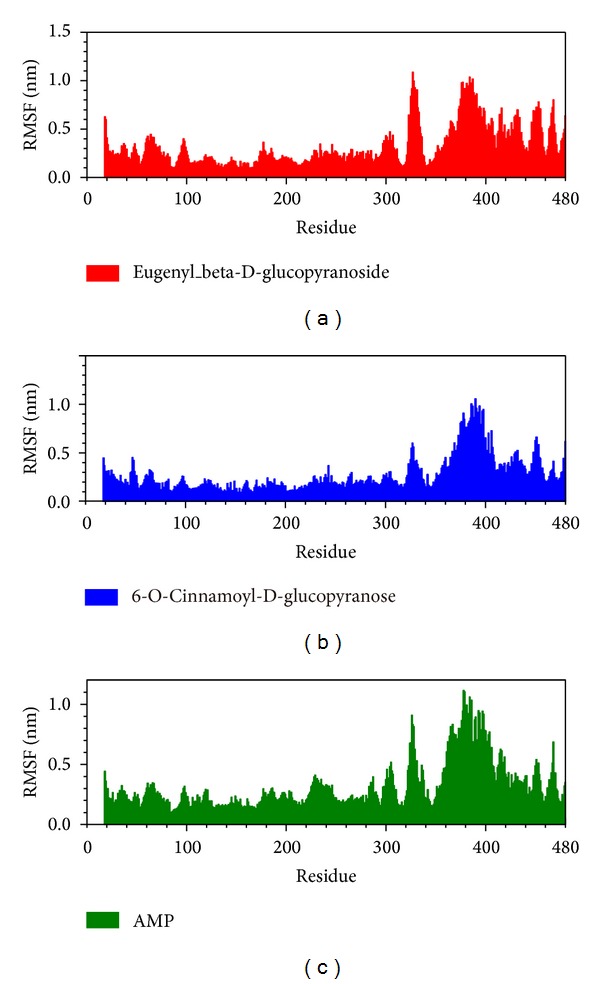
Root mean square fluctuation (RMSF) for (a) eugenyl_beta-D-glucopyranoside, (b) 6-O-cinnamoyl-D-glucopyranose, and (c) AMP.

**Figure 9:**
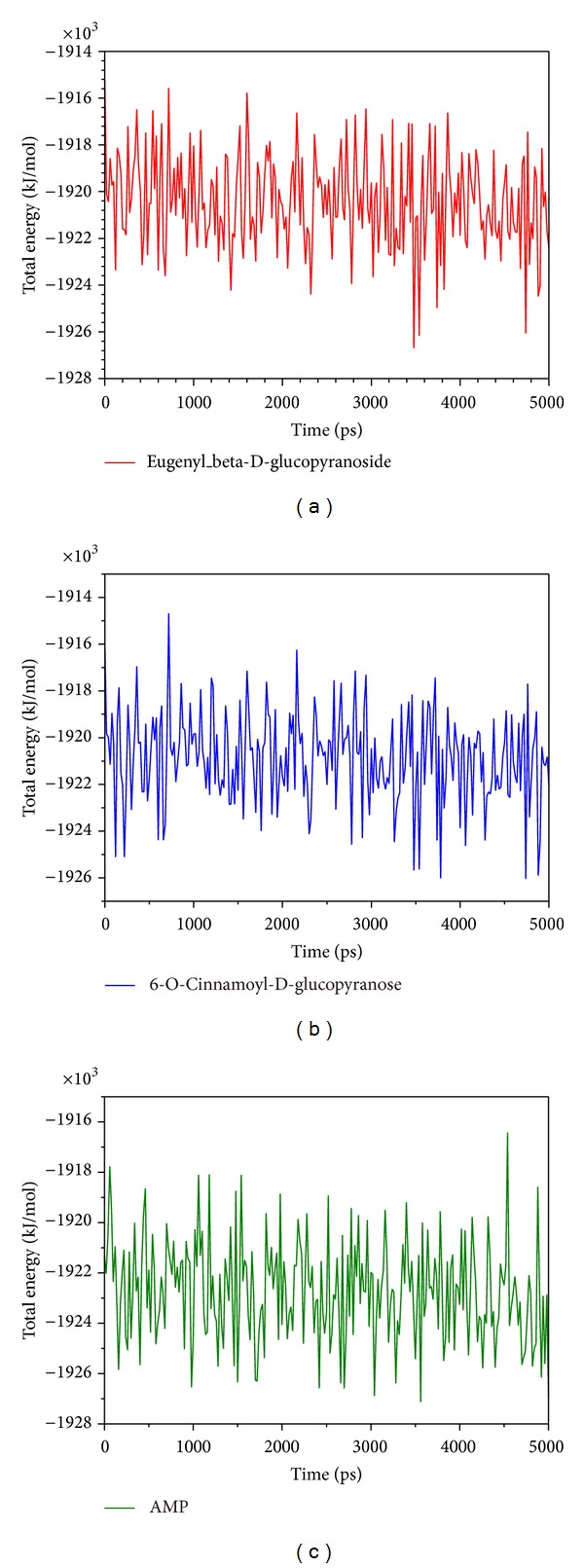
Total energy for (a) eugenyl_beta-D-glucopyranoside, (b) 6-O-cinnamoyl-D-glucopyranose, and (c) AMP.

**Figure 10:**
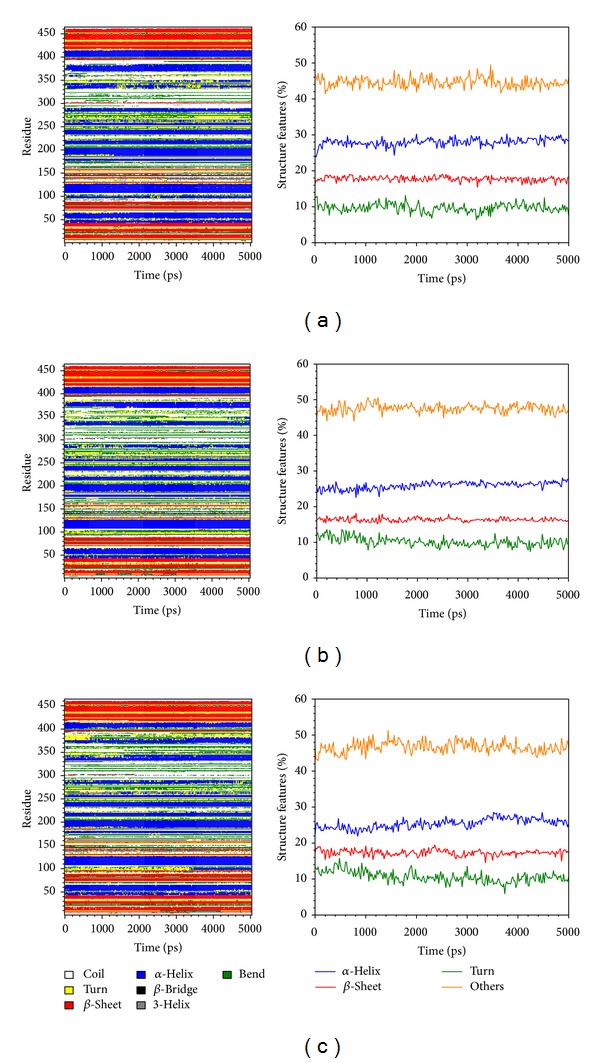
Database of secondary structure assignment (DSSP) and secondary structural component for (a) eugenyl_beta-D-glucopyranoside, (b) 6-O-cinnamoyl-D-glucopyranose, and (c) AMP.

**Figure 11:**
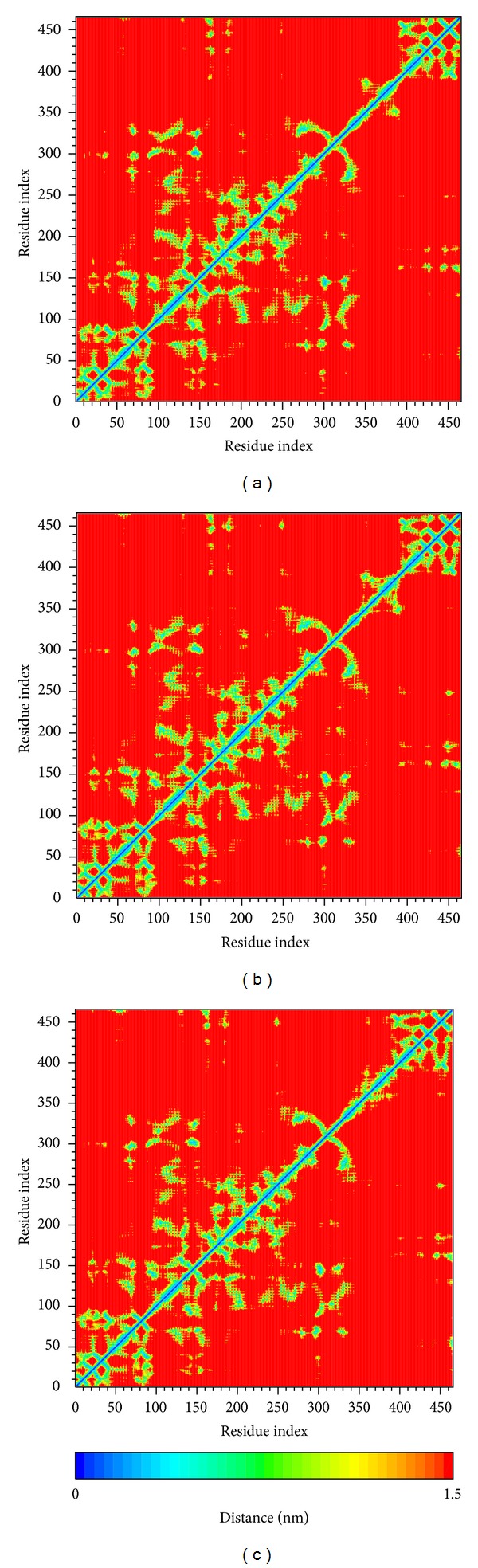
Matrices of the smallest distance of residues for (a) eugenyl_beta-D-glucopyranoside, (b) 6-O-cinnamoyl-D-glucopyranose, and (c) AMP.

Root mean square fluctuation (RMSF) showed the deviation of individual residue of the protein during MD. All the compounds had similar graphs with larger fluctuations at the range between residue 320 and residue 400 (Figure 8). AMP had a lower average total energy (−1923000 kJ/mol) than the 2 candidates (−1920000 and −1921000 kJ/mol) (Figure 9). Database of secondary structure assignment (DSSP) and secondary structural feature ratio variations of the protein was used to discuss the change of structural component during MD. All the candidates and the control had similar findings. Among the components of important second degree structure, the ratio of *α*-helix was higher than *β*-sheet, and *β*-sheet was higher than bend. The ratio of *α*-helix increased slightly, but the ratio of bend decreased comparatively (Figure 10). We utilized matrices of the smallest distance to find any variation of residue distance when the protein complexed with the ligand. There was not any significant difference compared to the candidates with the control (Figure 11).

Table 2 showed occupancy of H-bond between eugenyl_beta-D-glucopyranoside, 6-O-cinnamoyl-D-glucopyranose, and AMP with AMPK protein during MD. The formation of H-bond between eugenyl_beta-D-glucopyranoside with Asn138 and Asp151, 6-O-cinnamoyl-D-glucopyranose with Val18, Asp133, Lys135, and Asp151, and AMP with Gly19, Val90, and Glu94 was consistent with the results of docking. AMP also formed H-bond with Asp151, so we could conclude that Asp151 was key residue for top 2 TCM candidates and the control bound with AMPK.

**Figure 12:**
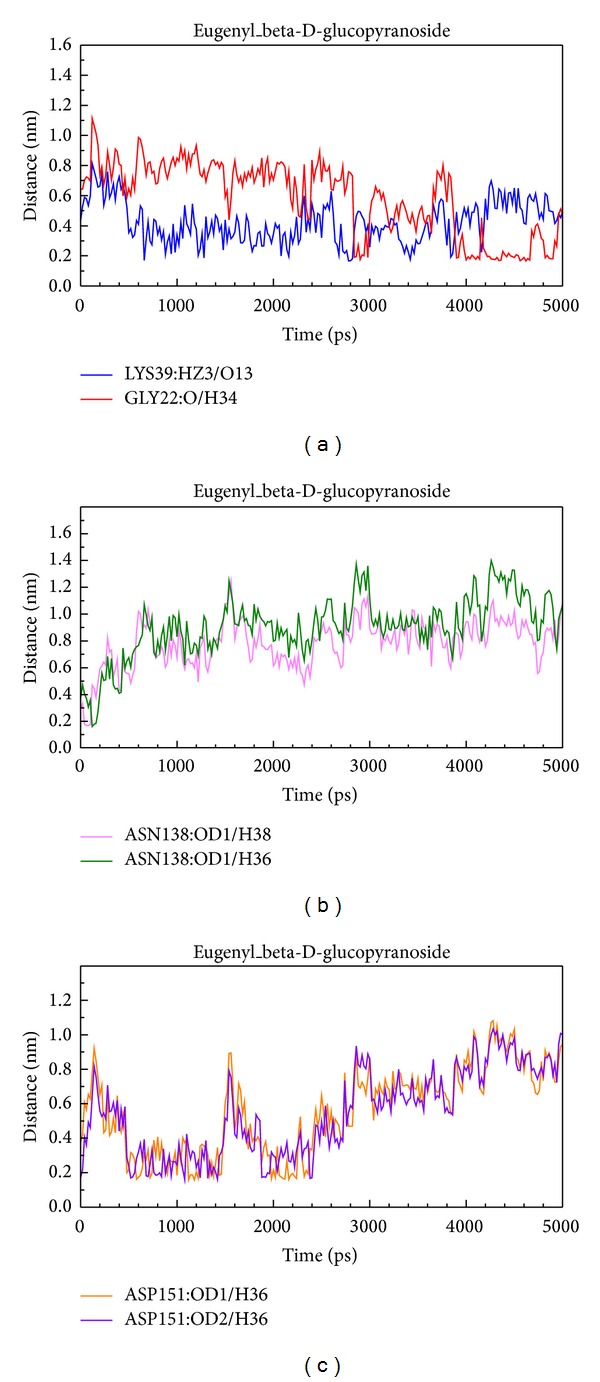
Distance of hydrogen bonds between eugenyl_beta-D-glucopyranoside and essential amino acids of AMPK.

We showed the distance of H-bonds between eugenyl_beta-D-glucopyranoside and essential amino acids of AMPK. The O13 of eugenyl_beta-D-glucopyranoside formed H-bond with Lys39 at early stage of MD, and H34 formed H-bond with Gly22 at late stage of MD. H36 and H38 formed H-bonds with Asn138 only at initial stage of MD. H36 formed H-bond with Asn151 at early stage of MD (Figure 12).

**Figure 13:**
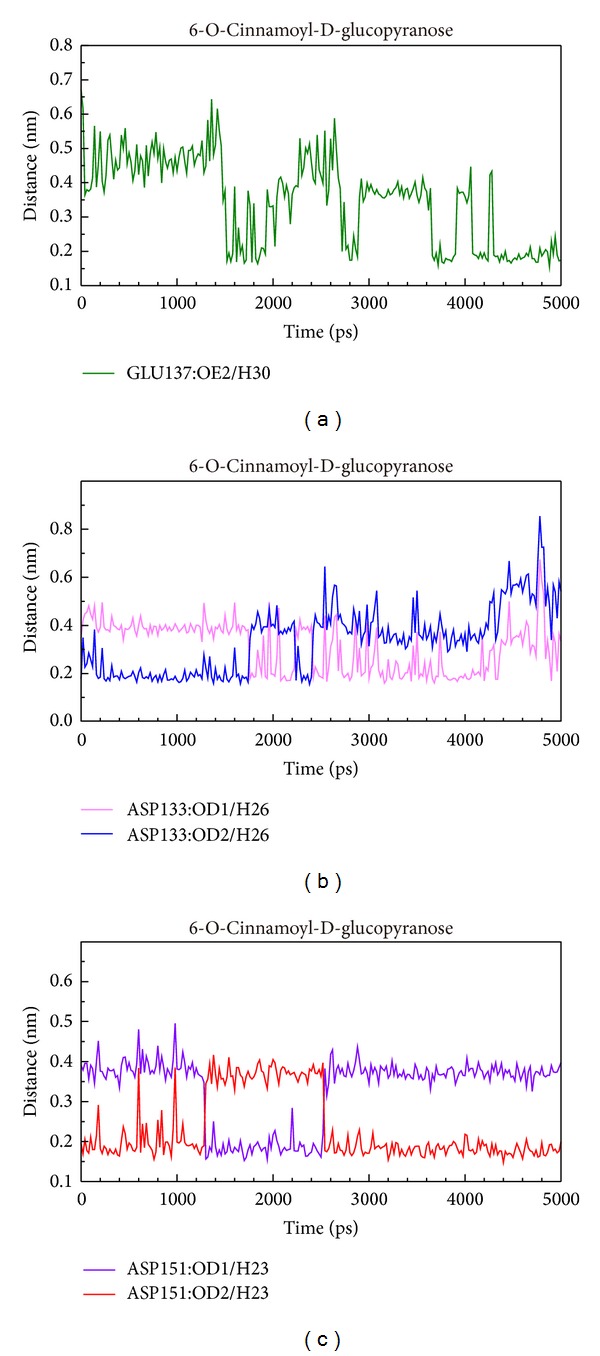
Distance of hydrogen bonds between 6-O-cinnamoyl-D-glucopyranose and essential amino acids of AMPK.

We showed distance of H-bonds between 6-O-cinnamoyl-D-glucopyranose and essential amino acids of AMPK. The H30 of 6-O-cinnamoyl-D-glucopyranose formed H-bond with Glu137 at late stage of MD. H26 formed H-bond with OD2 of Asp133 at early stage of MD and formed H-bond with OD1 of Asp133 at late stage of MD. H23 formed H-bond with OD2 of Asp151 at early and late stage of MD and formed H-bond with OD1 at middle stage of MD (Figure 13).

**Figure 14:**
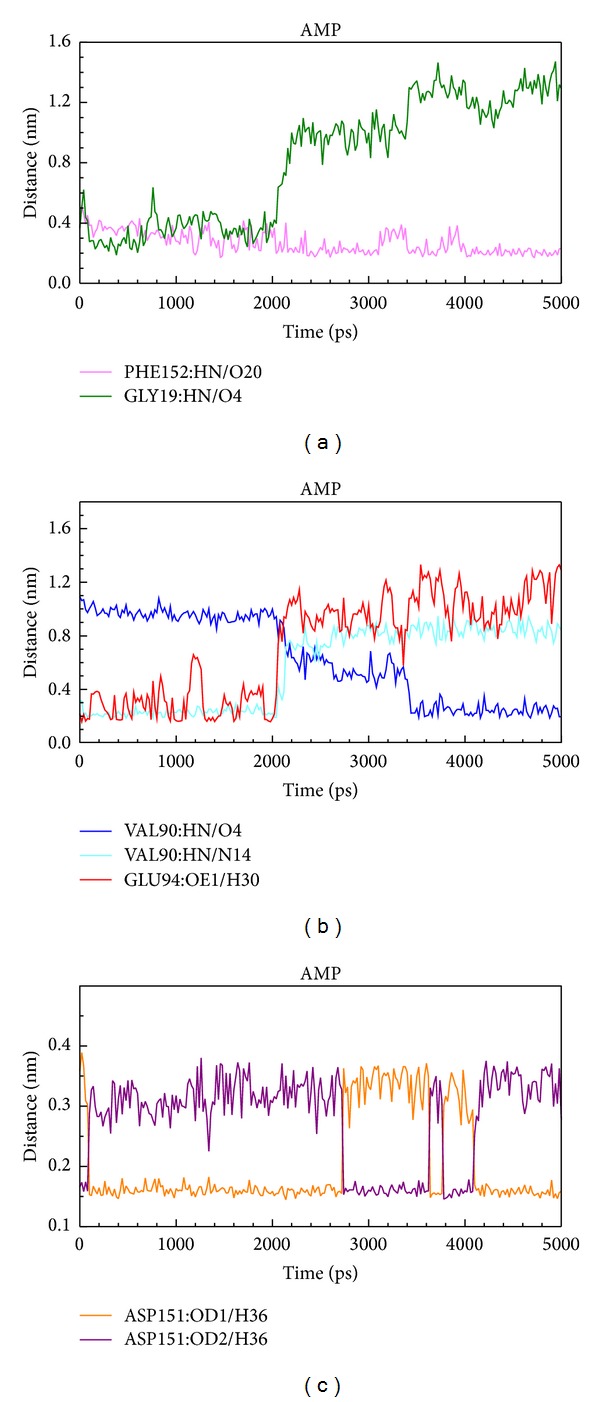
Distance of hydrogen bonds between AMP and essential amino acids of AMPK.

We showed distance of hydrogen bonds between AMP and essential amino acids of AMPK. The O4 of AMP formed H-bond with Gly19 at early stage of MD, and O20 formed H-bond with Phe152 at all stages of MD. H30 formed H-bond with Glu94 at early stage of MD. O4 formed H-bond with Val90 at early stage of MD, and N14 formed H-bond with Val90 at late stage of MD. H36 formed H-bond with OD1 of Asp151 at early and late stage of MD and formed H-bond with OD2 of Asp151 at middle stage of MD (Figure 14).

### 3.5. Ligand Pathway

**Figure 15:**
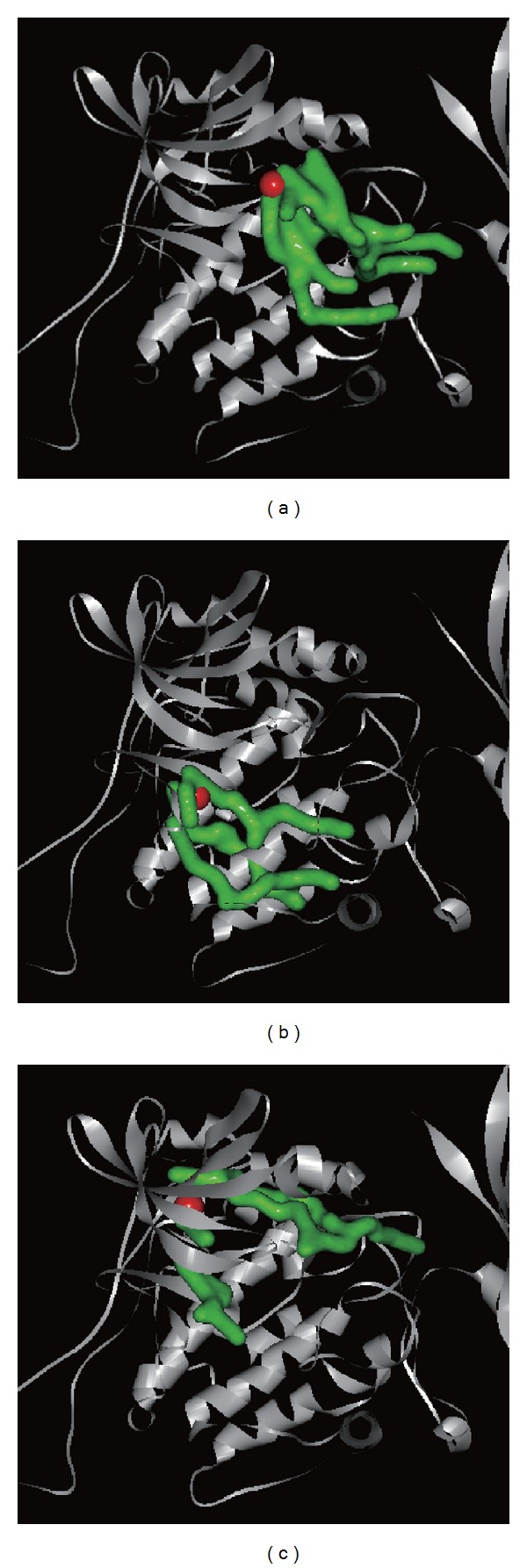
3D simulation of ligand pathway for (a) eugenyl_beta-D-glucopyranoside, (b) 6-O-cinnamoyl-D-glucopyranose, and (c) AMP bound with AMPK protein.

3D simulation of ligand pathway bound with AMPK helped us understand the process of combination. Eugenyl_beta-D-glucopyranoside, 6-O-cinnamoyl-D-glucopyranose, and AMP had different pathways bound with AMPK protein (Figure 15).

## 4. Discussion

### 4.1. Compound Database

The TCM small compounds for this study were from the world's largest TCM Database@Taiwan (http://tcm.cmu.edu.tw/). The TCM Database contained 453 kinds of herb plants, animals, and minerals. They consist of more than 20000 pure compounds. The TCM Database is a useful and precious treasury for exploring the mystery of traditional Chinese medicine.

### 4.2. Homology Modeling

Because the catalytic *α* subunit is the most important for all the three subunits of AMPK, we selected human AMPK (Q13131, *α* subunit) and rat templates (2Y94, *α* subunit) for homology modeling. The high percentage of identity and similarity of sequence alignment between Q13131 and 2Y94 indicated that the sequence alignment was reliable. The high percentage of residues in the favored and allowed area indicated that the AMPK-modeled structure was reasonable. The Verify scores of most residues were positive which indicated that the AMPK-modeled structure was reliable. We estimated that the negative values from residue 290 to residue 360 might be attributed to loss of reasonable structure from residue 311 to residue 341 illustrated in Figure 1.

### 4.3. Structure-Based Virtual Screening

Whether absorption level of ADMET, Dock scores, PLP1, PLP2 or PMF, all the top 7 TCM compounds were better than the control (AMP). Absorption level of ADMET from 0 to 3 means good to very low absorption. We selected the first 2 compounds, eugenyl_beta-D-glucopyranoside and 6-O-cinnamoyl-D-glucopyranose, as candidates due to their good absorption level. Both eugenyl_beta-D-glucopyranoside and 6-O-cinnamoyl-D-glucopyranose formed H-bond with Asp151. Both eugenyl_beta-D-glucopyranoside and AMP formed H-bond with Glu94. The main residues bound for the candidates and the control were located from Val18 to Asp151. The results mean that loss of reasonable structure from residue 311 to residue 341 did not disturb us for adopting the AMPK-modeled structure.

### 4.4. Molecular Dynamics (MD) Simulation

By integrating the figures of RMSD, Gyrate, and SAS, individual structure of the ligand and the protein underwent certain extent change during MD. This finding was essential for explanation of AMPK activation. Activation of AMPK followed its conformational change. Further analysis of RMSD, eugenyl_beta-D-glucopyranoside, 6-O-cinnamoyl-D-glucopyranose, and the control (AMP) had first larger deviation at 1600 ps, 1450 ps, and 2000 ps, which means that all the compounds could induce conformational change of AMPK. By further analysis of Gyrate and SAS, 6-O-cinnamoyl-D-glucopyranose had the largest ligand Gyrate and SAS values, but eugenyl_beta-D-glucopyranoside had the largest protein Gyrate and SAS values. We speculated that both the candidates could induce different kinds of conformational change of AMPK protein.

All the compounds had larger fluctuations at the range between residue 320 and residue 400 in RMSF. The larger fluctuations were not reliable due to loss of reasonable structure from residue 311 to residue 341. Based on the similar graph in RMSF and total energy, we could speculate that eugenyl_beta-D-glucopyranoside, 6-O-cinnamoyl-D-glucopyranose, and the control (AMP) had the same ability to activate AMPK. Our hypothesis was further approved by DSSP and smallest distance matrices. The structure of AMPK had similar change when it complexed with the 2 candidates and the control. Eugenyl_beta-D-glucopyranoside, 6-O-cinnamoyl-D-glucopyranose, and the control had the ability to induce AMPK conformational change.

According to occupancy of H-bond between eugenyl_beta-D-glucopyranoside, 6-O-cinnamoyl-D-glucopyranose, and AMP with AMPK protein, we could conclude that Asp151 was key residue for top 2 TCM candidates and the control bound with AMPK. By further analysis of distance of H-bonds between eugenyl_beta-D-glucopyranoside and essential amino acids of AMPK, the compound formed H-bond with Asp151 only at early stage of MD. At late stage of MD, the compound formed H-bond with Gly22 instead. By further analysis of distance of H-bonds between 6-O-cinnamoyl-D-glucopyranose and essential amino acids of AMPK, the compound formed H-bond with Asp151 at all stages of MD. By further analysis of distance of H-bonds between AMP and essential amino acids of AMPK, the compound formed H-bond with Asp151 at all stages of MD. Interestingly, H36 of eugenyl_beta-D-glucopyranoside formed H-bond with OD1 and OD2 of ASP151; H36 of AMP also formed H-bond with OD1 and OD2 of ASP151, but their figure patterns were quite different. However, H23 of 6-O-cinnamoyl-D-glucopyranose formed H-bond with OD1 and OD2 of ASP151 and the figure pattern was similar to that of AMP. We could confirm that all the candidates and the control formed stable complexes with AMPK. In addition, all the candidates and the control induced conformational change of AMPK due to changing position of H-bonds.

## 5. Conclusion

AMPK was a hot issue in the past decade because it plays an important role in sensing cellular energy or nutrient status. AMPK can regularize cell growth by the way of pseudostarvation leading to autophagy. Thus AMPK has become a great target for drug design of tumor inhibition. Activation of AMPK followed its conformational change. We tried to find potential compounds that could bind to AMPK by virtual screening of the world's largest TCM Database (http://tcm.cmu.edu.tw/) and had the ability to activate the function of AMPK. We selected eugenyl_beta-D-glucopyranoside and 6-O-cinnamoyl-D-glucopyranose as candidates for further investigation. Through the methods of MD simulation consisting of RMSD, Gyrate, SAS, RMSF, total energy, DSSP, smallest distance matrices, occupancy and distance of H-bonds, and ligand pathway, we could conclude that the candidates and the control (AMP) formed stable complexes with AMPK. Asp151 was key residue for the candidates and the control bound with AMPK. These compounds also had the ability to induce AMPK conformational change. Thus eugenyl_beta-D-glucopyranoside and 6-O-cinnamoyl-D-glucopyranose had the qualification to be AMPK agonist.

## Figures and Tables

**Table 1:** Top 7 candidates of scoring function from TCM Database@Taiwan screening. (ADMET_absorption level 0 to 3: good to very low absorption.)

Name	ADMET_Absorption_Level	Dock score	PLP1	PLP2	PMF
Eugenyl_beta-D-glucopyranoside	0	58.932	73.12	78.35	66.59
6-O-Cinnamoyl-D-glucopyranose	0	60.305	72.41	70.61	66.65
Loroglossin	1	69.903	74.94	71.77	72.83
Eleutheroside B	1	66.207	92.33	93.2	72.04
Quercetin-3-O-glucopyranoside	1	62.949	85.39	87.14	66.12
Synapic aldehyde 4-O-bata-D-glucopyranoside	1	62.262	94.7	94.75	73.44
(−)-9alpha-Hydroxysophocarpine	1	59.13	80.89	74.13	63.9

AMP	3	57.025	72.1	69.07	62.96

**Table 2:** Occupancy of H-bond between eugenyl_beta-D-glucopyranoside, 6-O-cinnamoyl-D-glucopyranose, and AMP with AMPK protein.

Ligand	H-bond	Ligand	Amino acid	Occupancy (%)
		Atom		
Eugenyl_beta-D-glucopyranoside	1	O15	THR20:HN	23.51%
	2	H38	THR20:OG1	14.34%
	3	O15	PHE21:HN	13.55%
	4	H34	GLY22:O	20.32%
	5	O13	LYS39:HZ3	19.92%
	6	H36	ASN138:OD1	1.59%
	7	H38	ASN138:OD1	1.99%
	8	H34	ASP151:OD1	14.34%
	9	H36	ASP151:OD1	20.32%
	10	H38	ASP151:OD1	7.17%
	11	H36	ASP151:OD2	21.51%

6-O-Cinnamoyl-D-glucopyranose	1	O22	VAL18:HN	16.33%
	2	O22	GLY19:HN	21.51%
	3	H26	ASP133:OD1	43.43%
	4	H28	ASP133:OD1	30.28%
	5	H26	ASP133:OD2	37.85%
	6	H28	ASP133:OD2	41.43%
	7	O6	LYS135:HZ3	15.54%
	8	H30	GLU137:OE1	27.49%
	9	H30	GLU137:OE2	33.07%
	10	H23	ASP151:OD1	24.70%
	11	H23	ASP151:OD2	64.94%

AMP	1	O4	GLY19:HN	13.94%
	2	O4	VAL90:HN	28.29%
	3	O6	VAL90:HN	11.55%
	4	N14	VAL90:HN	40.24%
	5	H30	VAL90:O	0.80%
	6	O6	GLU94:HN	10.76%
	7	H28	GLU94:OE1	6.77%
	8	H30	GLU94:OE1	22.31%
	9	H28	GLU94:OE2	4.38%
	10	H30	GLU94:OE2	19.92%
	11	H36	ASP151:OD1	77.29%
	12	H36	ASP151:OD2	47.81%
	13	O20	PHE152:HN	64.54%
